# Metabolic responses of adult lion’s paw scallops *Nodipecten subnodosus* exposed to acute hyperthermia in relation to seasonal reproductive effort

**DOI:** 10.1038/s41598-020-59242-6

**Published:** 2020-02-12

**Authors:** Rosa L. Salgado-García, Edouard Kraffe, Claudia I. Maytorena-Verdugo, Alma R. Rivera-Camacho, M. Teresa Sicard, Marcial Arellano-Martínez, Ilie S. Racotta

**Affiliations:** 1 Centro Interdisciplinario de Ciencias Marinas (CICIMAR), Instituto Politécnico Nacional (IPN), Av. Instituto Politécnico Nacional s/n Col, Playa Palo de Santa Rita Apdo. Postal 592, C. P. 23096 La Paz, B.C.S. Mexico; 2 Centro de Investigaciones Biológicas del Noroeste, S.C. (CIBNOR), Av. Instituto Politécnico Nacional 195, Playa Palo de Santa Rita Sur, C.P. 23096 La Paz, B.C.S. Mexico; 3 Univ Brest, CNRS, IRD, Ifremer, LEMAR, F-29280 Plouzane, France

**Keywords:** Ecology, Physiology

## Abstract

In marine ectotherms, reproduction is an energetically expensive process that affects their thermal window tolerance. For most species, the impacts of hyperthermia during gametogenesis have still not been addressed. Our aim was to assess the metabolic response of adult *Nodipecten subnodosus* scallops to thermal challenges at early development (spring) and advanced gonad maturation (summer). Scallops collected in both seasons were exposed to acute hyperthermia (26 and 30 °C, 24 h), maintaining a group of scallops at acclimation temperature (22 °C) as a control condition. During the summer, relatively low activity of hexokinase (HK), as well as low levels of ATP and GTP were found in the adductor muscle, suggesting a shift in energy investment for reproduction, although arginine phosphate (ArgP) levels were higher in summer scallops. Hyperthermia (30 °C) induced an increased energy expenditure reflected by a transitory enhanced oxygen consumption (VO_2_) and relatively high activities of HK and arginine kinase (AK). Moreover, a slight decrease in adenylic energy charge (AEC) was partially compensated by a decrease in ArgP. An increase in nucleotide by-products inosine monophosphate (IMP) and hypoxanthine (HX) indicated a thermal stress at 30 °C. Some of the responses to acute hyperthermia were more pronounced at advanced maturation stages (summer scallops), indicating a possible lack of energy balance, with possible implications in animals challenged to global warming scenario.

## Introduction

With the ongoing rise in global ocean temperatures and the increasing variability of seawater temperatures, the capability to acquire (ventilation) and supply (circulation) oxygen to tissues can limit the physiological activities and capabilities of ectotherms. Reproduction is an energetically expensive process that can affect the thermal tolerance thresholds of organisms^1–6^. Thermal tolerance studies have been generally conducted on juveniles, with few studies assessing the impacts of hyperthermia on the metabolic responses of adults during periods of high reproductive effort^7^.

In scallops, gonad growth is frequently but not always dependent on energy reserve mobilisation from the adductor muscle, in line with the reproductive strategy of the species, as well as on particular environmental conditions^8,9^. Gonad development can even impair tissue functionality, as shown by reduced muscular energy-related activity^10–12^, as well as shifts in metabolic enzyme kinetics^13–15^.

Molluscs use various strategies involving energy-rich phosphate compounds and anaerobic pathways when faced with hyperthermia to maintain an appropriate energy flux if the demand exceeds the tissue oxygen supply to the mitochondria^16^. In addition, animals can undergo metabolic depression to reduce energy demand and restore processes to increase the probability of survival under extreme conditions^5^. In scallops, a reduction in aerobic scope first affects active tissues such as the digestive gland and adductor muscle^17^. Overall, reproduction and hyperthermia can affect muscle functionality, and therefore, a synergistic influence of both factors is expected, compromising overall performance and even survival. In several mollusc species, summer mortality events occur when the temperature approaches the critical thermal limit of a species, mainly affecting mature and post spawning animals, in which any increase in energy demand may lead to metabolic stress^18^. Nevertheless, massive die-off events result from the interactions between several biotic and abiotic factors, such as the presence of pathogens, temperature, hypoxia, and reproductive effort^19,20^. Warmer summers can provide favourable conditions for spawning and settlement, but severe temperature increases can cause metabolic depression and can reduce immunological capability^20,21^. In addition, increased temperature events can inhibit gametogenesis, reduce gamete quality, and cause both gamete reabsorption and disruption in spawning time^22^.

Lion’s paw scallops *N*. *subnodosus* (Sowerby, 1835) are hermaphroditic species distributed along the Pacific coast from Peru to northern Mexico and had been harvested as a high-quality food product for several decades in the Ojo de Liebre Lagoon, Baja California Sur (27°55′N 114°20′W), until natural populations started to collapse in 2010^23^. Although some hypotheses linked to valve deformities, dark colouration of the adductor muscle, decreased reproductive output and poor health conditions have been suggested, there is still no explanation for the massive mortality in this area^24^. An increase in summer mortality events has also been reported in cultured populations of *N*. *subnodosus*^25^, although interactions with other endogenous (*e*.*g*., reproductive effort) or exogenous (*e*.*g*., hyperthermia, hypoxia, food availability and pathogens) factors were not investigated. Thermal tolerance of juvenile lion’s paw scallops have been determined, with a lethal temperature of 32 °C^26^, but no studies on the effects of high temperature in combination with a related biological trait, such as reproductive effort, on adult scallops exist.

We hypothesise that adult *N*. *subnodosus* scallops undergo severe metabolic constraints during their peak reproductive periods, challenging their capabilities to cope with hyperthermia. In this context, the objective of this study was to assess the metabolic responses in the adductor muscle of adult scallops from the Pacific coast of Baja California after they had been exposed to realistic acute hyperthermia challenges during early and advanced gonad growth stages. To this end, we compared scallops sampled from a culture system in Baja California Sur at two periods of the year showing two distinct gonad development stages and exposed the scallops to three different temperatures (22, 26 and 30 °C) for 24 h. Oxygen consumption was measured to evaluate the thermal tolerance of the respiratory capacities of the individual animals. We also examined the adductor muscle metabolic responses in terms of the cellular energy state and enzyme activities.

## Materials and Methods

### Collection site

Magdalena Bay is a coastal lagoon on the Pacific coast of the Baja California Peninsula, Mexico, between 25°10′10″N–112°11′24″W and 24°26′24″N–113°33′00″W. Thermal profile data of the sea surface temperature (SST) of the lagoon (Aqua MODIS, NPP, 4 km, daytime, 11 microns, 2003-present, monthly composite) and chlorophyll-*a* data (mg L) (Aqua MODIS, NPP, L3SMI, global, 4 km, science quality, 2003-present, monthly composite) were obtained for a decade (2006–2016) from the NOAA (National Oceanic and Atmospheric Administration, US). Additionally, *in situ* SST records were obtained from the culture site (<2 m depth) every 30 min using a HOBO pendant data logger (Onset UA-002-64, US) during April-May (spring) and June-July (summer) 2016 (Supplementary Fig. 1).

### Animal sampling and maintenance

*N*. *subnodosus* specimens were collected from a suspended culture system (<2 m depth) inside the Magdalena Bay lagoon during the reproductive season previously reported by Arellano-Martínez *et al*.^27^. We selected two stages of the annual reproductive cycle in this region: initial development (on May 21^st^; spring) and advanced development (on July 8^th^; summer). At each sampling date, sixty scallops were randomly collected, and the gonads of 10 scallops were dissected, and then individually fixed in Davidson’s solution for further histological analysis^27^. The remaining scallops (n = 50) were carefully placed in a polyurethane box that contained aerated seawater from the culture site and transported to the Centro de Investigaciones Biológicas del Noroeste (CIBNOR) in La Paz, Mexico. During transport (2–3 h), the temperature was monitored using a HOBO data logger (Onset UA-002-64, US) and maintained at 22 ± 1 °C by adding precooled seawater to standardise the transport and acclimatisation temperature on both sampling dates. Upon arrival, the animals were placed in a 150 L water tank and acclimated for eight days at 22 ± 1 °C, under a controlled photoperiod (12 L: 12 D). The water temperature was continuously monitored with a HOBO data logger (Onset UA-002-64, US), while the water salinity (35–36 ppm; Extech Instruments, Waltham, MA, US) and *p*O_2_ (90% air saturation; Microx TX2, Presens, Germany) were measured daily. During acclimation, the animals were fed a microalgae diet consisting of *Isochrysis galbana* and *Chaetoceros calcitrans* (90,000 cells mL^−1^, 1:1). The food consumption was monitored daily using a particle counter (multisizer, Beckman, US).

### Experimental design and oxygen consumption measurements

The experimental design was partially based on the SST data recorded at the collection site during the spring and summer (Supplementary Fig. 1), which resulted in the scallops being subjected to circadian thermal fluctuations between 19.9 and 25.1 °C and between 20.0 and 27.8 °C one week before the sampling day, respectively. After acclimation, the animals collected each season (spring and summer) were exposed to three experimental treatments: one group was exposed to 22 °C, which is the optimum temperature for juvenile growth^26^, the second group was exposed to 26 °C, and the third group was exposed to 30 °C, which is close to the upper lethal temperature reported for *N*. *subnodosus* juveniles (32 °C)^26^.

For each experimental group, ten animals were randomly selected, cleaned from epibionts and individually placed in open flow-through glass respiration chambers (60 mL min^−1^) connected to a header tank, under experimental conditions. The scallops were placed in the chamber at the acclimation temperature (22 °C) and fasted for 12 h before the thermal challenges were started. Water temperature was then gradually increased (1 °C h^−1^) from the acclimation temperature to one of the two higher target temperatures (26 or 30 °C), after which the temperature was held constant for 24 h. The temperature was continuously monitored using data loggers (Onset UA-002-64, US). Water dissolved oxygen levels were monitored using oxygen microsensors (Microx TX2, Presens, Germany) and were kept above 70% air saturation in all experiments.

Once the target temperature was reached, individual oxygen consumption was measured at 0, 6, 12, 18 and 24 h. The dissolved oxygen levels (% air saturation) were measured using microsensors (Microx TX2, Presens, Germany) connected to the outflow of each chamber (60 mL min^−1^). A chamber containing an empty shell was used as a blank to correct background respiration caused by microorganisms^28^. The oxygen microsensors were calibrated at each experimental temperature using Na_2_SO_3_ (Fermont, Mexico) saturated water for 0% air saturation and fully aerated seawater for 100% saturation. The oxygen level (percent air saturation) was corrected to the temperature and salinity-specific oxygen capacity of water^29^. Individual oxygen consumption rates (VO_2_) were measured for 2 min by switching between animal chambers at each sampling time. The individual oxygen consumption was calculated as follows: *VO*_2_ = (Δ*VO*_2_ × *V*_*f1*_)/*M*, where ΔVO_2_ is the oxygen consumption of an animal (μmol O_2_ g^−1^ h^−1^) corrected according to the oxygen level in the control respirometer *VO*_2_ = (*VO*_2*control*_ − *VO*_2*animal*_), V_fl_ is the flow rate (L h^−1^), and M is the total soft tissue wet mass (g).

### Tissue collection

After 24 h of thermal challenge, the animals were removed from the respiration chambers, and their entire soft tissue biomass was quickly weighed, sampled and immediately frozen in liquid nitrogen. The shells were cleaned, dried, and weighed, after which they were measured with a digital calliper (CD-6CS, Mitutoyo, Japan). The condition index (CI) was calculated as $$[total\,soft\,tissue\,wet\,weight\,(g)/dry\,shell\,weight(g)]\times $$$$100$$^30^, and the adductor muscle index (AMI) was calculated as $$[adductor\,muscle\,wet\,weight(g)/total\,soft\,tissue\,wet\,weight\,(g)]\,\times 100$$^30^.

### Gonadal development

As a quantitative criterion of gonad development, the gonadosomatic index (GSI) was calculated as the percentage of gonad weight to remaining tissue weight rather than total tissue weight using the following formula: $$GSI:\,[gonads\,wet\,weight(g)/(total\,soft\,tissue\,wet\,weight(g)-$$$$gonads\,wet\,weight(g))]\times 100$$, considering that the GSI is affected by changes in mass of either gonadal or somatic tissue as the proportion of gonad wet weight in relation to the total somatic tissue wet weight^8^. In addition, the gonadal mass index (GMI) was calculated as the proportion of gonad mass in relation to standardised shell height of scallops used in this study (77.55 mm). The allometric relationship between gonad size and body size can be expressed as *Y* = *aH*^*b*^, where Y is the gonad mass in grams, H is the shell height in millimetres for each season, a is the intercept, and b is the exponent (allometric coefficient), all of which were obtained from log (base 10)-transformed values of gonad mass and shell height to achieve linearity and homogeneity of variances via the following equation: $$\log \,Y=\,\log \,a+b\,\log \,H$$^31,32^.

For a qualitative description of gonadal development, histological analysis was performed on 10 scallops collected during both seasons (spring and summer). Each gonad portion was dehydrated via an alcohol series of increasing concentrations (70–100%), cleared with Hemo-De and embedded in Paraplast-Xtra using a microtome, 4 µm-thick sections were obtained and stained with Harris hematoxylin and eosin (H&E) (Humason, 1979) according to histological methods for gonad phases: (I) undifferentiated, characterised by the absence of gametic cells; (II) early development, with expanded follicles and contain oogonia or spermatogonia attached to the follicle wall; (III) late development, characterised by an increase in mature gametes; (IV) maturation (or ripe), with follicles nearly full of free post vitellogenic oocytes in the lumen; V) spawning or partially spawned, with variable quantities of follicles that are partially or totally empty; (VI) spent or post spawning, which corresponds to the recovery phase after spawning; and (VII) resorption, which includes phagocytosis of residual oocytes^9,27^. Gonadic development images (40X) were obtained using a microscope (Leica DM4B, Germany) connected to a digital camera (Leica, DMC2900, Germany) in conjunction with LAS V software 4.12.

### Metabolite extraction and quantification

Frozen samples of adductor muscle were ground to a fine powder with a ball mill mixer (MM400, Retsch, Germany) that was precooled with liquid nitrogen. Nucleotides within the ground adductor muscle samples (100 mg) were extracted and processed according to the methods described by Moal *et al*.^33^, with modifications as described by Robles-Romo *et al*.^34^. Acidic extracts (200 µl) were neutralised with a mixture of dichloromethane and trioctylamine (5:1 *v/v*), after which they were passed through a 0.2 µm filter and then maintained at −80 °C until further analysis. The nucleotides were separated by ion-pairing reversed-phase HPLC (model 1100, Agilent Technologies, Palo Alto, CA) with a Hyperclone ODS C18 column (150 × 4.6 mm, 3 µm particle size; Phenomenex, Torrance, CA) connected to a C18 guard column (40 × 3 mm; Phenomenex, Torrance, CA). Separation was carried out in a mobile phase consisting of 0.15 M sodium phosphate monobasic (H_2_NaO_4_P), 3 mM tetrabutylammonium (Sigma-Aldrich, Merck, St. Louis, MO) and 8% methanol at pH 6.0, which was adjusted with 5 N NaOH. Nucleotide signals were detected at 254 nm at 0.8 mL min^−1^ for 22 min. Nucleotide identification was performed using a mixture of standards of ATP, ADP, AMP, GTP, IMP and HX (Sigma-Aldrich, Merck, St. Louis, MO) at known concentrations. The adenylate energy charge (AEC) was estimated according to the methods of Atkinson^35^ as follows: [(ATP + 0.5ADP)/(ATP + ADP + AMP)].

Arginine phosphate (ArgP) was analysed from the same neutralised extracts used for nucleotide quantification according to the methods of Viant *et al*.^36^, with modifications as described by Robles-Romo *et al*.^34^ by reversed-phase HPLC in conjunction with a SpheroClone NH_2_ column (250 × 4.6 mm, 5 µm particle size; Phenomenex, Torrance, CA) connected to a C18 security guard cartridge (40 × 3 mm; Phenomenex, Torrance, CA). The separation was performed at a rate of 1.0 mL min^−1^ for 18 min in a mobile phase consisting of 0.02 M KH_2_PO_4_ buffer (Sigma-Aldrich, Merck, St. Louis, MO) and acetonitrile (72:28) adjusted to pH 2.6 with 5 M H_3_PO_4_. ArgP identification and quantification were performed at 254 nm using a commercial standard with a known concentration (Santa Cruz Biotechnology, Santa Cruz, CA). All HPLC-grade reagents were purchased from Fermont, Mexico, and all solutions used were fresh and filtered using a 0.45 µm nylon membrane.

### Enzyme activity assays

Activities of metabolic enzymes, hexokinase (HK), pyruvate kinase (PK), cytochrome c oxidase (CCO), citrate synthase (CS), lactate dehydrogenase (LDH), octopine dehydrogenase (ODH), and arginine kinase (AK) were quantified in adductor muscle samples (50 mg) from animals exposed to 22 °C and hyperthermia at 30 °C.

Frozen powder tissue samples were homogenised with specific buffers (1:10, *w/v*) for each enzyme (Table 1) using a Polytron device (model PT 6100D, Kinematica AG, Switzerland) at 180 *g* for 30 s at 4 °C and then sonicated (Q125-110, Qsonica Sonicators, Newtown, CT) at time intervals of 10 s (30% output, 4 °C). All samples were analysed during the first hour after homogenisation. Triplicates of each sample were analysed for enzyme activity at 24 °C using a microplate reader (Varioskan Lux, Thermo Fisher Scientific, Finland). Enzyme activity was expressed as units per gram of tissue wet weight (U g^−1^), where U represents the amount of micromoles of substrate converted to product per minute. All chemicals used were purchased from Sigma-Aldrich Merck, St. Louis, MO. The reaction conditions for the enzyme assays are as follows:

**Table 1:** Homogenisation and assay conditions for the quantification of metabolic enzymes in adductor muscle of adult *N*. *subnodosus* scallops.

Enzyme	Tissue homogenization buffer	Reference
Hexokinase (HK)	50 mM Tris-HCl, 1 mM EDTA, 2 mM MgCl_2_, 2 mM DTT, pH 7.4.	^57^
Pyruvate kinase (PK)	50 mM Imidazole, 1 mM EDTA, 0.1% Triton X100, pH 7.2.	^58^
Cytochrome c oxidase (CCO)	100 mM Tris-HCl, 5 mM EDTA, 2 mM PMSF, 0.1% Triton X100, pH 7.2.	^14^
Citrate synthase (CS)	100 mM Tris-HCl, 5 mM EDTA, pH 7.2.	^59^
Lactate dehydrogenase (LDH)	50 mM Imidazole-HCl, 1 mM EDTA, 0.1% Triton X100, pH 6.6.	^60^
Octopine dehydrogenase (ODH)	50 mM Imidazole-HCl, 1 mM EDTA, 0.1% Triton X100, pH 6.6.	^60^
Arginine kinase (AK)	50 mM Imidazole, 1 mM EDTA, 0.1% Triton X100, pH 7.2.	^58^

*Hexokinase* (EC 2.7.1.1): 75 mM Tris-HCl, 7.5 mM MgCl_2_, 0.8 mM EDTA, 3 mM KCl, 2 mM DTT, 2.5 mM ATP, 1 mM glucose, 0.4 mM NADP, 1 U mL^−1^ G6PDH, pH 7.3. Abs.: 340 nm. K: 10 min. εNADH = 6.22 mmol L^−1^ cm^−1^.

*Pyruvate kinase* (EC 2.7.1.40): 50 mM imidazole-HCl, 60 mM MgCl_2_, 2.5 mM ADP, 0.15 mM NADH, 1 U mL^−1^ LDH, 2.5 mM PEP, pH 7.2. Abs.: 340 nm. K: 14 min. εNADH = 6.22 mmol L^−1^ cm^−1^.

*Cytochrome c oxidase* (EC 1.9.3.1): 50 mM phosphate, 50 µM cytochrome c, pH 7.0. Abs.: 550 nm. K: 10 min. εCyt c = 29.5 mmol L^−1^ cm^−1^.

*Citrate synthase* (EC 2.3.3.1): 100 mM Tris-HCl, 0.1 mM acetyl-CoA, 0.1 mM DNTB, 1 mM oxaloacetate, pH 8.0. Abs.: 412 nm. K: 14 min. εTNB = 13.6 mmol L^−1^ cm^−1^.

*Lactate dehydrogenase* (EC 1.1.1.27): 50 mM imidazole-HCl, 2.5 mM sodium pyruvate, 0.1 mM NADH, pH 6.6. Abs.: 340 nm. K: 10 min. εNADH = 6.22 mmol L^−1^ cm^−1^.

*Octopine dehydrogenase* (EC 1.5.1.11): 50 mM imidazole-HCl, 2.5 mM sodium pyruvate, 3 mM L-arginine, 0.1 mM NADH, pH 6.6. Abs.: 340 nm. K: 10 min. εNADH = 6.22 mmol L^−1^ cm^−1^.

*Arginine kinase* (EC 2.7.3.3): 50 mM imidazole-HCl, 10 mM MgCl_2_, 10 mM ATP, 1 mM PEP, 0.40 mM NADPH, 1 U mL^−1^ PK, 1 U mL^−1^ LDH, pH 7.2. Abs.: 340 nm. K: 14 min. εNADH = 6.22 mmol L^−1^ cm^−1^.

### Statistical analysis

All variables were analysed for normality (Bartlett test) and homoscedasticity (Levene test). Arcsine and logarithmic transformations were performed if necessary before statistical analysis. One-way repeated measures ANOVA was used to evaluate the effects of thermal challenge (between subjects) over time (within subjects) on the oxygen consumption rate in each season. The Bonferroni test was applied for mean comparisons using the oxygen consumption rate values (control) before the temperature challenge. Two-way ANOVA was used to test the effects of season (spring and summer) and temperature (22, 26 and 30 °C) on nucleotide concentrations and enzymatic activities. Only when a significant interaction was observed individual means for the season plus temperature combinations were compared (Tukey’s HSD test). Otherwise, global means within each factor were compared and indicated in the text. All the statistics and graphics were analysed by Statistica version 7.0 (StatSoft, Tulsa, OK) and GraphPad Prism version 8.0 for Windows (GraphPad, La Jolla CA), respectively. In all cases, statistical significance was accepted at *p* < 0.05.

## Results

### Gonad and adductor muscle indices

**Figure 1:**
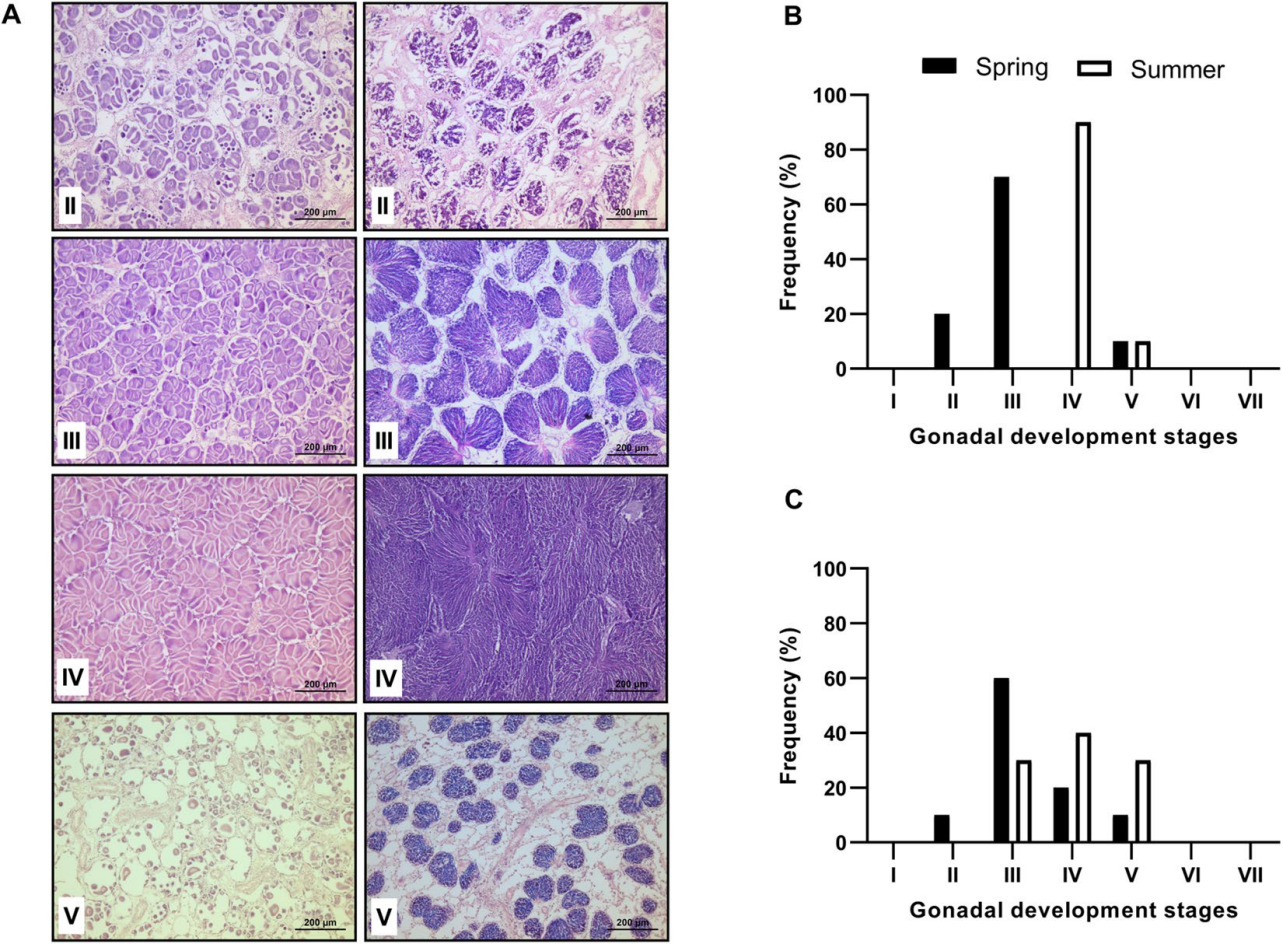
Gonad histology and gametogenesis of *N*. *subnodosus* scallops. (**A**) Histological sections of female (left) and male (right) gonads showing the following development stages detected in individuals collected during both periods: early development (II), late development (III), maturation (IV), and spawning (V). Conversely, the following stages were not observed in the scallops analyzed: undifferentiated (I), postspawning (VI) and resorption (VII). Frequency of gonad development stages in female (**B**) and male (**C**) gonadic sections of animals collected in the spring (black) and summer (white). The frequency values are expressed as the percentage of animals by group. (n = 10 scallops for each season).

An increase in gonad size (GSI, GMI) and more advanced gonadal development were identified in summer scallops, in accordance with previous studies analysing reproduction of *N*. *subnodosus* at Magdalena Bay and in the Ojo de Liebre Lagoon on the Baja California Peninsula^27,37^. The female part of the hermaphroditic gonad developed more in the summer (10% spawned and 90% ripe) than in the spring (20% early development, 70% late development and 10% spawned). The same trend was observed for the male gonad part, with an increase in the proportion of ripe and spawned gonads and a decrease in late development gonads (Fig. 1A–C).

Both gonad indices were also significantly higher in summer scallops (GSI: 11.2 ± 0.9, GMI: 4.5 ± 0.3) than in spring scallops (GSI: 7.6 ± 0.5; GMI: 3.0 ± 0.2). Temperature challenge significantly increased the adductor muscle index at 30 °C compared to 22 and 26 °C, regardless of the season (Table 2).

**Table 2:** Biometric variables of adult *N*. *subnodosus* scallops exposed to elevated temperatures (26 and 30 °C) during early development (spring) and advanced gonad maturation (summer) stages.

Variable	Spring			Summer			S	T	S × T
	22 °C	26 °C	30 °C	22 °C	26 °C	30 °C	*p* < 0.05	*p* < 0.05	*p* < 0.05
Tissues wet weight (g)	45.0 ± 2.9	42.8 ± 2.4	43.1 ± 3.8	48.7 ± 4.7	42.4 ± 2.6	48.5 ± 3.4	NS	NS	NS
Shell length (mm)	79.5 ± 2.7	79.8 ± 2.3	78.4 ± 2.3	77.0 ± 2.1	73.0 ± 1.7	77.6 ± 2.1	NS	NS	NS
Shell height (mm)	79.0 ± 2.2	76.6 ± 1.9	78.5 ± 1.9	77.4 ± 2.3	75.6 ± 1.4	78.8 ± 2.0	NS	NS	NS
Condition Index (CI, %)	68.3 ± 6.8	66.5 ± 2.3	59.3 ± 2.5	67.4 ± 2.6	66.5 ± 2.3	65.2 ± 2.1	NS	NS	NS
Adductor Muscle Index (AMI, %)	38.1 ± 1.0	36.9 ± 9.0	39.7 ± 0.8	36.2 ± 0.7	37.1 ± 0.6	38.6 ± 0.8	NS	***p*** < **0**.**01**	NS
Gonadosomatic Index (GSI, %)	6.0 ± 0.6	8.0 ± 1.2	8.1 ± 1.0	10.3 ± 1.1	11.1 ± 1.5	12.1 ± 2.0	***p*** < **0**.**05**	NS	NS
Gonadal Mass Index (GMI)	2.5 ± 0.32	3.3 ± 0.35	3.3 ± 0.039	4.4 ± 0.41	4.3 ± 0.55	3.33 ± 0.78	***p*** < **0**.**05**	NS	NS

Data are expressed as mean value ± standard error of mean. Two-way ANOVA results between seasons (S) and temperatures (T) are shown in last columns (NS: not significant).

### Oxygen consumption rate (VO_2_)

**Figure 2:**
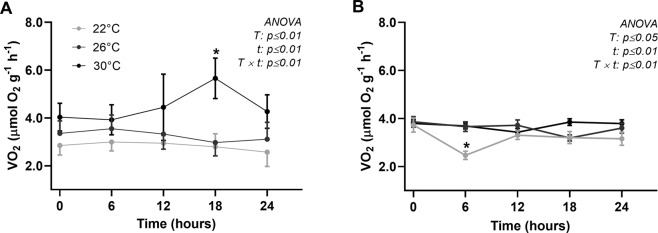
Oxygen consumption rate of *N*. *subnodosus* scallops (VO_2_, µmol O_2_, g^−1^ h^−1^) exposed to acute thermal challenges (22, 26 and 30 °C) in the spring (**A**) and summer (**B**). Two-way ANOVA repeated mesures between temperature conditions (T) and time (t) (0, 6, 12, 18 and 24 h) are inserted in Figure. The asterisks represent significant difference (p < 0.05) against t 0 by Bonferroni test. The values are expressed as the means ± SEs of the means, n = 10 scallops for each group.

In the spring, the animals at 22 and 26 °C maintained a stable VO_2_, whereas the animals at 30 °C presented an increased VO_2,_ with maximum values attained at 18 h of thermal exposure, followed by a return to baseline values at 24 h (Fig. 2A). In the summer, the VO_2_ remained stable overall, with a slight but significant decrease at 6 h of exposure in the control group (22 °C) (Fig. 2B).

### Nucleotides, AEC and ArgP

**Figure 3:**
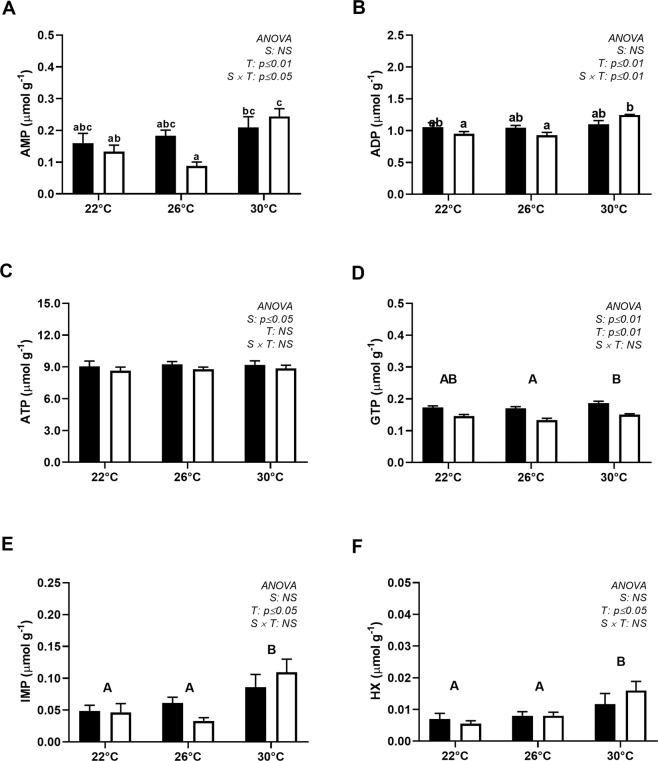
Nucleotide composition of the adductor muscle of *N*. *subnodosus* scallops exposed to acute thermal challenges (22, 26 and 30 °C) in the spring (black) and summer (white). (**A**) Adenosine monophosphate (AMP, µmol g^−1^). (**B**) Adenosine diphosphate (ADP, µmol g^−1^). (**C**) Adenosine triphosphate (ATP, µmol g^−1^). (**D**) Guanosine triphosphate (GTP, µmol g^−1^). (**E**) Inosine monophosphate (IMP, µmol g^−1^). (**F**) Hypoxanthine (HX, µmol g^−1^). The two-way ANOVA results are shown in the figure. Following Tukey’s post hoc mean comparisons, the different lowercase letters represent significant interactions between season (S) and temperature (T), whereas the different capital letters indicate significant differences between temperatures when a significant main effect of this factor was observed (see the Materials and Methods). n = 7 scallops at each temperature × season combination. The values are expressed as the means ± SEs of the means.

AMP, ADP, IMP and HX increased in animals exposed to 30 °C, although for AMP and ADP, such effects were observed only in the summer, as shown by a significant interaction effect (Fig. 3A–F). ATP was not affected by the thermal challenge, while GTP was significantly lower at 26 °C than at 30 °C (Fig. 3C,D).

**Figure 4:**
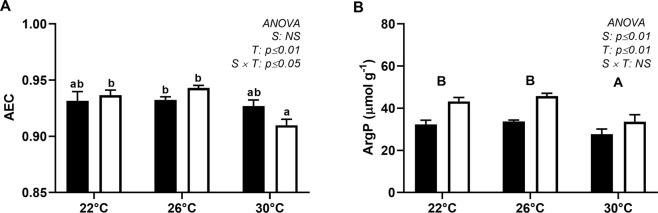
Energy content of the adductor muscle of *N*. *subnodosus* scallops. (**A**) Adenylate energy charge (AEC) and (**B**) Arginine phosphate (ArgP, µmol g^−1^) levels in animals exposed to different thermal conditions (22, 26 and 30 °C) in the spring (black) and summer (white). See the legend of Fig. 3 for the relevant statistical data.

Only scallops collected in summer showed a significant decrease in AEC values at 30 °C compared to 22 and 26 °C (Fig. 4A). Summer scallops presented higher levels of ArgP than those collected in the spring, regardless of the thermal challenge (Fig. 4B). Exposure to 30 °C significantly decreased the ArgP level during both sampling periods. In the spring, ArgP decreased from 32.3 ± 2.0 and 33.6 ± 0.7 µmol g^−1^ at 22 °C and 26 °C, respectively, to 27.6 ± 2.5 µmol g^−1^ at 30 °C, whereas in the summer, ArgP decreased from 43.2 ± 1.9 and 45.7 ± 1.3 µmol g^−1^, at 22 °C and 26 °C, respectively, to 33.5 ± 3.4 µmol g^−1^ at 30 °C (Fig. 4B).

### Metabolic enzymes

**Figure 5:**
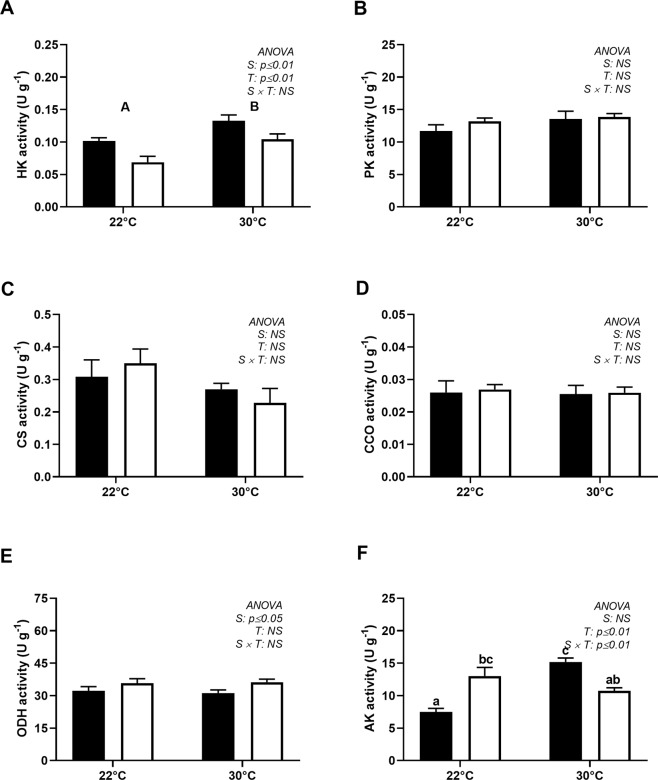
Activities of aerobic and anaerobic enzymes in the adductor muscle of *N*. *subnodosus* scallops exposed to control conditions (22 °C) and acute hyperthermia challenge (30 °C) in the spring (black) and summer (white). (**A**) Hexokinase activity (HK, U g^−1^). (**B**) Pyruvate kinase activity (PK, U g^−1^). (**C**) Citrate synthase activity (CS, U g^−1^). (**D**) Cytochrome c oxidase activity (CCO, U g^−1^). (**E**) Octopine dehydrogenase activity (ODH, U g^−1^). (**F**) Arginine kinase activity (AK, U g^−1^). See the legend of Fig. 3 for the relevant statistical data.

**Figure 6:**
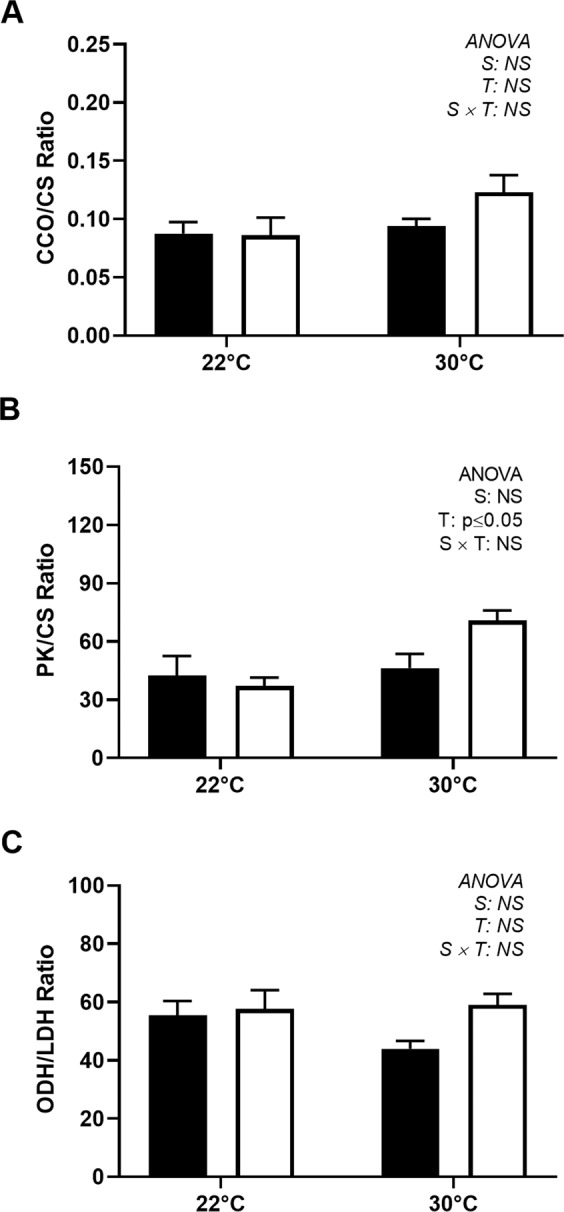
Ratios of enzyme activities in the adductor muscle of *N*. *subnodosus* scallops exposed to control conditions (22 °C) and acute hyperthermia challenge (30 °C) in the spring (black) and summer (white). (**A**) CCO/CS ratio. (**B**) PK/CS ratio. (**C**) ODH/LDH ratio See Fig. 3 for the relevant statistical data.

The HK activity in the spring was significantly higher than that in summer and increased during both seasons when the animals were challenged at 30 °C (Fig. 5A). The activity of PK was similar across temperatures and seasons (Fig. 5B), while that of CS presented a slight but nonsignificant decrease at 30 °C (Fig. 5C), resulting in a increased PK/CS ratio in the animals exposed to hyperthermia (30 °C), especially in the spring (Fig. 6B). The activity of CCO and the CCO/CS ratio were not affected by the thermal challenge (Figs. 5D and 6A). The activity of ODH was 50 times higher than that of LDH in the adductor muscle of *N*. *subnodosus* (Fig. 6C). The LDH activity was similar across both seasons and thermal conditions (0.66 ± 0.02 U g^−1^), but the ODH activity was slightly but significantly higher in the summer than in the spring (Fig. 5E); such a difference did not result in a significant effect on the ODH/LDH ratio (Fig. 6C). The AK activity displayed a marked seasonal sensitivity to temperature, as it increased by 42% in the spring following temperature challenge at 30 °C but decreased by 17% in animals challenged in the summer (Fig. 5F).

## Discussion

To identify potential metabolic constraints to which adult scallops experiencing maturation could be exposed during elevated temperature, this work analysed seasonal patterns of cellular energy status in *N*. *subnodosus* adults exposed to acute hyperthermia under experimental conditions at different stages of gonad development. The temperature challenge of 30 °C was chosen based on the thermal tolerance of populations of *N*. *subnodosus* from Baja California Sur, whose lethal temperature (96 h-LD_50_) ranged between 28 and 31.4 °C^26^, which also corroborated realistic maximum temperatures that occurred within diurnal thermal fluctuations in this area (Supplementary Fig. 1).

The influence of reproductive activity on muscle reserves and capabilities has been widely addressed in molluscs [for reviews, see^5,8^]. A shift in aerobic power from basic functions such as swimming performance is clearly evidenced by lower capabilities of adductor muscles to sustain an escape response, including recovery from bursts of anaerobic activity in several pectinids [for a review, see^5^]. In the present study, the decrease in ATP and GTP levels found in the adductor muscle of scallops that had reached advanced gonadal maturation in the summer supports the relatively high energy demand imposed by reproduction. In turn, a relatively low HK activity could indicate a reduced glucose uptake and phosphorylation concomitant with muscle glycogen mobilisation for lipid accumulation in gonads^8^. On the other hand, high levels of ArgP in the adductor muscle of summer scallops seem to be related to other factors influencing the availability of this short-term high-energy reserve. It should be noted that the scallops sampled in the summer and challenged to 22 and 26 °C have levels greater than 45 µmol g^−1^ that represent five times the pool of ATP (9 µmol g^−1^) observed in the same scallops. In addition, such levels were higher than those observed for this species in the field (27 µmol g^−1^) at the same locality and season (unpublished results), as well as in other species of scallops from Antarctic and temperate habitats (20 to 30 µmol g^−1^)^38^. Therefore, scallops apparently have a higher capability to store ArgP in the summer than in the spring when exposed to laboratory conditions and fed *ad libitum*, which in turn could reflect different metabolic efficiencies between scallops collected during each season. Additional studies are needed to confirm and establish the basis of such seasonal related metabolic capabilities and to explore the reliance on exogenous (environmental conditions) and/or endogenous factors (reproductive effort).

Although it is well known that metabolic rates increase with acute increases in temperature, summer scallops displayed unchanged VO_2_ values throughout the 24 h hyperthermia challenge, whereas a transient increase in VO_2_ was observed in response to the 30 °C challenge in spring scallops. Such apparent seasonal sensitivity to thermal challenge related to reproductive activity (discussed below) reflects compensatory mechanisms that counteract the variation in the metabolic rate in response to seasonal temperature changes^39,40^.

A decrease in AEC and ArgP together with an increase in IMP and HX following thermal challenge could reflect an increase in energy use (ATP and ArgP) not fully compensated by its rate of synthesis^16^. Increased IMP, and particularly HX, normally occurs under postmortem conditions as a consequence of nucleotide degradation^41^, although increased levels of IMP can occur in live animals under some stress conditions^42^. The IMP concentration in adult scallops exposed to 30 °C (0.1 µmol g^−1^) reached half that found in the postmortem adductor muscle of *N*. *subnodosus* (0.2 µmol g^−1^), while the concentration of HX reached only 0.01 µmol g^−1^ compared to 0.51 µmol g^−1^ under one-day-in ice postmortem conditions^43^. Further implications for increased periods of hyperthermia on scallop “quality” based on IMP and HX levels should be analysed, as several other indices of postmortem quality of meat (muscle hardness, protein and lipid content, specific amino acid composition) were lower in lion’s paw scallops collected in the summer than in other seasons^44^. An increase in IMP and HX nucleotides could also indicate a signal of stress conditions under acute hyperthermia. Seasonal effects on the response to temperature challenge were observed for the increase in AMP and ADP and the concomitant decrease in AEC at 30 °C in scallops collected in the summer. A decrease in AEC indicates an inability to maintain an energy balance at the cellular level and therefore represents a powerful regulatory signal to activate the production of ATP from its accumulated hydrolysis products, AMP and ADP^45^. In turn, a decreased AEC is indicative of stress in response to sublethal environmental changes^46^, although the lowest levels attained in the present study (≥0.90) are above the levels considered indicative of mild (0.5–0.75) or severe (~0.5) stress^47^. Buffering AEC could be achieved by short-term mobilization of ArgP, as shown in several situations of high ATP demand, such as intense muscular activity^34,48^, hypoxia^49^ and hyperthermia^50^. Such a metabolic strategy seemed to occur in adult scallops in which ATP content was not altered at the expense of decreased levels of ArgP at 30 °C. This effect was more accentuated in summer scallops (10 µmol g^−1^) than in spring scallops (5 µmol g^−1^), considering the difference between the levels of ArgP at 30 °C compared to lower temperatures (22 and 26 °C). This indicates a possible shift of energy balance affected by gonadal development and environmental conditions.

Ectotherms can respond to both short-term and chronic temperature changes with quantitative and/or qualitative adjustments in enzyme activity^51^. Several mechanisms, such as isozyme expression, mitochondrial volume and density, and lipid composition of the mitochondrial membrane, can explain seasonal or even circadian temperature acclimation [for review, see^53^]. In the scallops in this study, up- or down regulated compensatory adjustment related to hyperthermia and reproductive stage was also observed. The aerobic capability of the scallop’s adductor muscle is affected under thermal challenges and then gradually adjusts itself to achieve compensation^52,53^. In the present study, the activities of CCO and CS were not affected by the temperature challenge (30 °C) under laboratory conditions, indicating a lack of compensatory changes in the mitochondrial properties/activities over the 24 h exposure time. In contrast, temperature challenge stimulated HK activity during the early and advanced gonadal stages (spring and summer), suggesting an increased capability of glucose phosphorylation in line with increased metabolic (mainly glycolytic) flux. Activities of PK, another important regulated glycolysis enzyme, displayed no significant differences with respect to the temperature challenge or gonad maturation stage. However, the ratio of PK/CS was relatively low in the scallops exposed to acute temperature stress at 30 °C in the spring, indicating that an enhanced anaerobic glycolytic potential is stimulated by acute hyperthermia during early gonad maturation stages^54,55^. This probably involves preparation for the transition from aerobic to anaerobic energy metabolism^51^. Accordingly, ODH activity in the adductor muscles of *N*. *subnodosus* reached higher values at the most advanced stages of gonad maturation (summer), which is related to reproductive investment in scallops^11,12^. Together with higher levels of ArgP in summer scallops discussed below, this suggests that an enhanced anaerobic capability closely matches spawning events, which would constitute an adaptation in preparation for high-energy-demanding periods such as spawning and for increased temperatures. The ODH activity is temperature independent, which has been interpreted as an indication of a temperature-regulating mechanism that enables the related enzymes to maintain levels of NAD, NADH and octopine constant and independent of temperature^51^. AK activity displayed a higher thermal sensitivity in the spring scallops, as evidenced by the significant increase in activity after the hyperthermia challenge (30 °C), whereas the summer scallops displayed no significant changes. This is likely related to the seasonal acclimation of the enzyme as the SST starts to increase (Supplementary Fig. 1) and could be related to the highest content of ArgP in the summer scallops. Previous studies in scallops have suggested that AK is stimulated by a decrease in ArgP content and by an increase of free arginine, ADP and Pi^38,56^. Thus, it seems likely that the decrease in ArgP and the simultaneous increase in ADP in the summer scallops exposed to 30 °C were counteracted by other factors probably related to their reproductive stages and the environmental conditions in the field during that period, as suggested on the basis of the levels of ArgP. Additional studies are needed to explore the direct seasonal influence in field scallops and the effects of acclimation to laboratory conditions such as being fed *ad libitum* and constant temperature.

## Conclusion

These results support the hypothesis that *N*. *subnodosus* scallops display a seasonal pattern in energy metabolism related to gonad maturation. The seasonal shifts in the energy state (AEC, ArgP), oxygen consumption rate and glycolysis activity (HK) in response to acute hyperthermia suggest that *N*. *subnodosus* adults exhibit different sensitivities to thermal conditions during gonad maturation. While this could negatively affect the energy supply and balance, the response observed in adult scallops suggested that *N*. *subnodosus* presents a high capability to respond to acute temperature stress in the range of its thermal window tolerance. Our study examined the capability of scallops to respond to an acute challenge of 24 h, these results lead to additional unknowns regarding the metabolic and energetic capability of the response and homeostasis under repeated exposure to acute hyperthermia scenarios and regarding the increasing variability in seawater temperature as another possible large event in future global warming scenarios.

## Supplementary information

Supplementary information.

## Data Availability

No datasets were generated or analysed during the current study.
