# Supplementary material for: Metabolic responses of adult lion’s paw scallops Nodipecten subnodosus exposed to acute hyperthermia in relation to seasonal reproductive effort
Source: Sci Rep. 2020 Feb 12;10:2449. doi: 10.1038/s41598-020-59242-6 (PMC7015932; doi:10.1038/s41598-020-59242-6)
Supplement: Supplementary file 1 — Supplementary information. [file 41598_2020_59242_MOESM1_ESM.pdf]

Metabolic responses of adult lion's paw scallops (*Nodipecten subnodosus*) exposed to acute hyperthermia in relation to seasonal reproductive effort

Salgado-García, Rosa L.[1, 2], Kraffe, Edouard[3], Maytorena-Verdugo, Claudia I.[2], Rivera-Camacho, Alma R.[1], Sicard, M. Teresa[2], Arellano-Martínez, Marcial[1], Racotta, Ilie S.[2*].

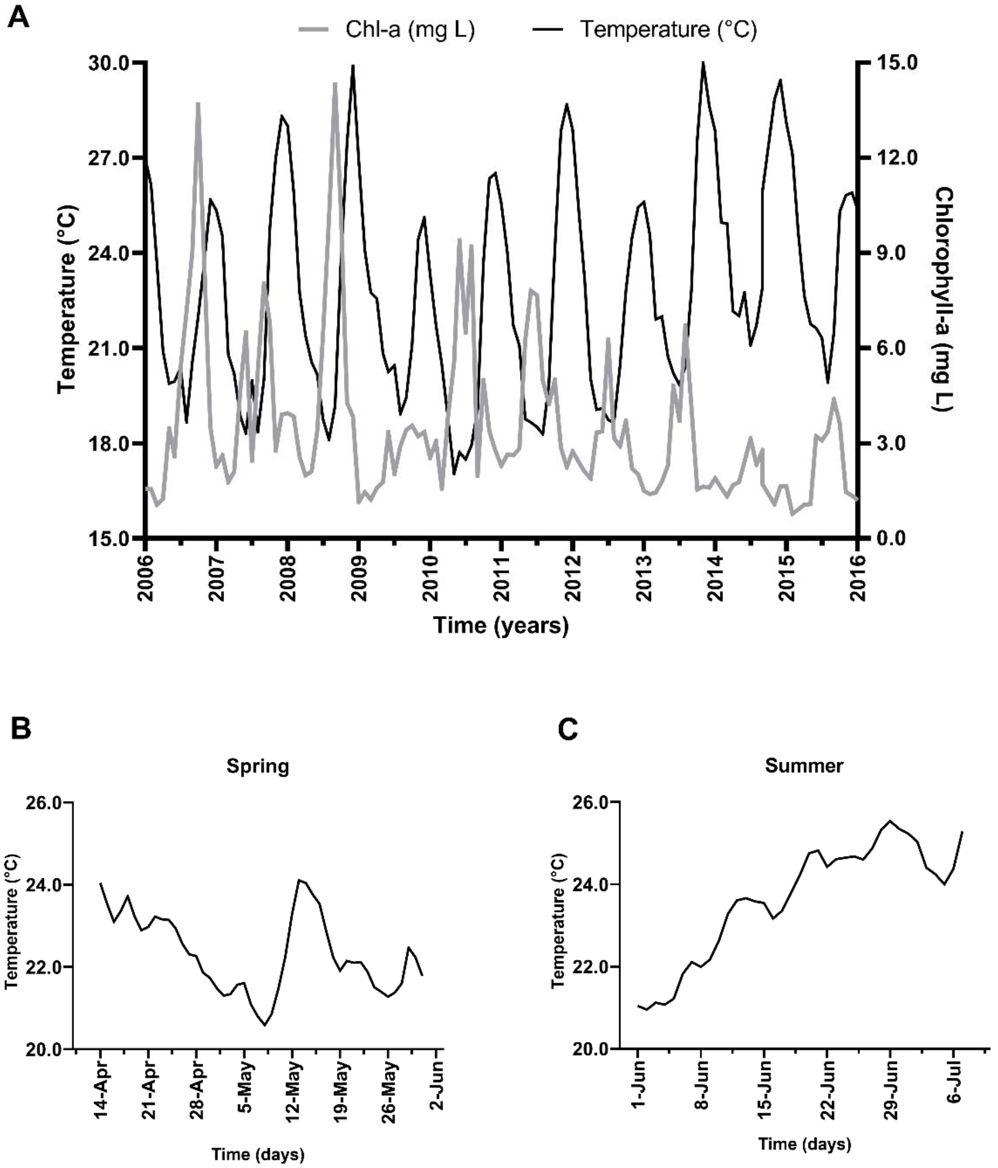

Supplementary Figure 1. Water temperature data and Chlorophyll-a in the Magdalena Bay lagoon. A. Superficial water temperature (°C) and chlorophyll a (mg L) from October 2006 to October 2016. B. Temperature data base registered daily in spring (April-May 2016) and C. summer (June-July 2016) in culture site of *N. subnodosus* in Magdalena Bay Lagoon.
